# Reversible Deactivation Radical Polymerization: From Polymer Network Synthesis to 3D Printing

**DOI:** 10.1002/advs.202003701

**Published:** 2021-01-21

**Authors:** Ali Bagheri, Christopher M. Fellows, Cyrille Boyer

**Affiliations:** ^1^ School of Science and Technology The University of New England Armidale NSW 2351 Australia; ^2^ Desalination Technologies Research Institute Al Jubail 31951 Kingdom of Saudi Arabia; ^3^ Centre for Advanced Macromolecular Design (CAMD) and Australian Centre for NanoMedicine (ACN) School of Chemical Engineering The University of New South Wales Sydney NSW 2052 Australia

**Keywords:** 3D printing, dormant initiating sites, photoactivated polymerization, polymer crosslinked networks, reversible deactivation radical polymerization, transformable materials

## Abstract

3D printing has changed the fabrication of advanced materials as it can provide customized and on‐demand 3D networks. However, 3D printing of polymer materials with the capacity to be transformed after printing remains a great challenge for engineers, material, and polymer scientists. Radical polymerization has been conventionally used in photopolymerization‐based 3D printing, as in the broader context of crosslinked polymer networks. Although this reaction pathway has shown great promise, it offers limited control over chain growth, chain architecture, and thus the final properties of the polymer networks. More fundamentally, radical polymerization produces dead polymer chains incapable of postpolymerization transformations. Alternatively, the application of reversible deactivation radical polymerization (RDRP) to polymer networks allows the tuning of network homogeneity and more importantly, enables the production of advanced materials containing dormant reactivatable species that can be used for subsequent processes in a postsynthetic stage. Consequently, the opportunities that (photoactivated) RDRP‐based networks offer have been leveraged through the novel concepts of structurally tailored and engineered macromolecular gels, living additive manufacturing and photoexpandable/transformable‐polymer networks. Herein, the advantages of RDRP‐based networks over irreversibly formed conventional networks are discussed.

## Graphical abstract

The application of reversible deactivation radical polymerization (RDRP) to polymer networks and 3D printing allows the tuning of network homogeneity and more importantly, enables the production/manufacturing of advanced materials containing dormant reactivatable species that can be used to change the material's properties in a postsynthetic/printing stage, a characteristic that is not achievable using conventional techniques.

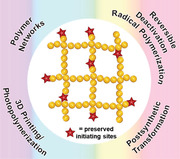

## 1 Introduction

Polymer networks can be formed in bulk, solution or using 3D printing and they can be classified into four main categories: thermosets, thermoplastics, elastomers, and gels.^[^ ^1^ ^]^ Recent advances in the field of polymer networks have conferred new properties, for instance, self‐healing, ultrahigh permanent porosity, and stimuli‐responsiveness. These properties have expanded the applications of polymer networks in energy harvesting/storages,^[^ ^2^ ^]^ coatings/adhesives,^[^ ^3^ ^]^ porous materials,^[^ ^4^ ^]^ biomedical materials,^[^ ^5^ ^]^ etc.

Radical polymerization has been the most widely used mechanism for the synthesis of polymer networks due to its broad monomer scope and functional group tolerance.^[^ ^6^ ^]^ However, conventional radical polymerization offers limited control over chain growth resulting in the production of dead polymer chains incapable of postfunctionalization and further chain extension. In contrast, reversible deactivation radical polymerization (RDRP)^[^ ^7^ ^]^ techniques such as nitroxide‐mediated polymerization, (NMP, also referred to as aminoxyl‐mediated radical polymerization),^[^ ^8^ ^]^ atom transfer radical polymerization (ATRP),^[^ ^9^ ^]^ and reversible addition–fragmentation chain transfer (RAFT)^[^ ^10^ ^]^ polymerization have the ability to reversibly deactivate propagating radial species, which allow control over polymer architectures. In addition to this benefit, one of the greatest opportunities provided by RDRP processes in the context of polymeric networks is the ability to produce advanced materials containing dormant reactivatable species capable of undergoing postsynthetic transformations which enable modification of the structure, functionality, and physicochemical and mechanical properties of a pristine network in a postsynthetic stage. Whereas early RDRP systems relied on thermal initiators to generate active species, other stimuli, and in particular, light, have been used to eliminate the dependence on the use of elevated temperature and confer spatial and temporal controls over polymerization.^[^ ^11^, ^12^, ^13^ ^]^ Photoactivated RDRP systems (photoRDRP) have showed promises in a variety of perspectives, such as, nanotechnology, nanomedicine, precision polymerization, etc.^[^ ^14^, ^15^, ^16^, ^17^, ^18^ ^]^ In addition, photoRDRP systems have been successfully exploited in both synthesis and postsynthesis modifications of crosslinked polymer networks. In the context of RDRP‐based networks, novel concepts of structurally tailored and engineered macromolecular (STEM) gels,^[^ ^19^ ^]^ living additive manufacturing (LAM)^[^ ^20^ ^]^ and photoexpandable/transformable‐polymer networks (PET‐PNs)^[^ ^21^ ^]^ have been recently emerged.

Inspired by the opportunities that photoRDRP has to offer, recently its application to 3D printing has been explored to facilitate 3D printing of networks with dormant initiating sites that can be used for postprinting modifications, e.g., postprinting polymerization initiated from the preserved RDRP functionalities.^[^ ^22^, ^23^, ^24^ ^]^ Taking one step back, 3D printing via photopolymerization provides a programmable pathway for the layer by layer fabrication of customized and on‐demand 3D networks with complex shapes and functionalities, and without wasting excess materials.^[^ ^25^, ^26^ ^]^ One could imagine that if a printed material has defects or some of its physicochemical/mechanical properties need minor alteration, postprinting modification could be a more viable approach rather than fabrication of a new object. It should be mentioned that 4D printing (fourth dimension being time) has been developed to enable printing of materials with the ability to change their shapes, sizes, and properties in response to an external stimulus such as temperature, water, pH, light, etc.^[^ ^27^, ^28^, ^29^, ^30^, ^31^, ^32^, ^33^, ^34^ ^]^ This ability is normally realized by integration of stimuli responsive functionalities into the resin formulations which can become a part of the printed network.^[^ ^31^ ^]^ The use of RDRP processes in 3D printing where RDRP agents can be used for further modification of 3D materials can be also considered as a subcategory of the 4D printing concept.

This review is therefore focused on the use of RDRP processes to produce crosslinked polymer networks. In Section 2, we briefly discuss the importance of polymer networks in advanced materials and their formation mechanisms. The benefits of using RDRP techniques in the polymer network synthesis are concisely discussed (Section 3). Photoactivation mechanisms in photoRDRP and their unique properties are also elaborated (Section 4). An overview of the recent developments in the field of (photo)RDRP‐based polymer networks are provided in Section 5. This review also highlights the application of photoRDRP in 3D printing together with current challenges that need to be overcome (Section 6).

## 2 Importance of Polymer Networks in Advanced Materials and their Formation Mechanisms. Why Do Polymer Networks Matter?

**Figure 1:**
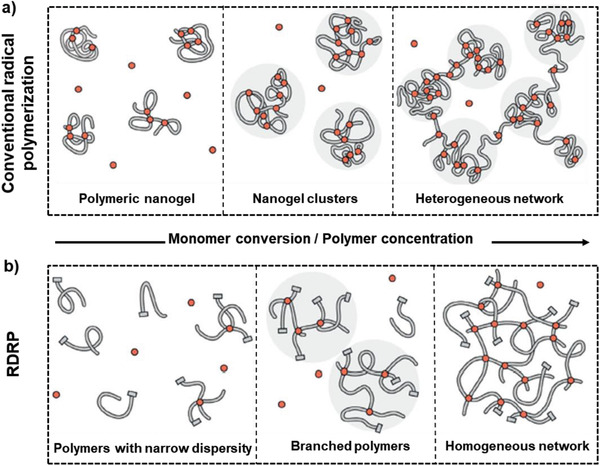
Different gelation processes using a) radical polymerization and b) reversible deactivation radical polymerization (RDRP) processes. In contrast to the heterogeneous networks synthesized via conventional radical polymerization, RDRP leads to the formation of more homogeneous networks. Reproduced with permission.^[^ ^1^ ^]^ Copyright 2020, John Wiley and Sons.

Polymer networks are materials composed of smaller components known as crosslinks and strands, which are connected via covalent or noncovalent interactions (**Figure** **1**). Properties of polymer networks rely on the composition of the crosslinks, strands, and topology. Majority of polymer networks can be categorized into four main groups: thermosets, elastomers, thermoplastics, and gels. We note that in all of these systems, intermediate species such as nanogels and microgels which are precursors to network formation are formed in the phase prior to gelation (pregelation). Polymer networks are known as thermosets if there is covalent bonding between crosslinks and strands, and the material is at a temperature below the glass transition temperature. Therefore, these materials are extremely rigid (Young's moduli of around 10^6^ kPa), insoluble and not reprocessable, unless a particular chemistry mechanism is applied to enable transformability/reprocessability of these materials.^[^ ^3^, ^35^ ^]^ Elastomers are polymer networks in which macromolecules are covalently bonded together or crosslinks are provided by physical entanglements between chains, which are not covalently linked. In contrast to thermosets, elastomers are used at temperatures above their glass transition temperature to offer soft materials (Young's moduli lower than 10^3^ kPa) with reversible stretchability. The third category of polymer networks is thermoplastics which are connected via intermolecular noncovalent interactions and are typically used below their glass transition temperature—this allows them, in principle, to be remolded, healed, and recycled. The last category of polymer networks is gels which can be formed from either covalent or supramolecular bonds. Gels are capable of swelling in liquid media and are typically soft with relatively low Young's moduli (typically less than 10 kPa). It should be mentioned that polymer networks categorized as thermosets, elastomers, and thermoplastics can be considered as gel if they meet the criteria specified for the gel category.

Polymeric networks play critical roles in both academic and industrial environments due to their unique properties, such as swellability, elasticity, porosity, and tunable mechanical stability, which were conventionally used in numerous applications including rubber products, cosmetics, sorbents, adhesives, membranes, coating materials, and food packaging. Throughout the years, as the field of polymer networks has evolved and advanced properties (such as self‐healing, conductivity, ultrahigh permanent porosity, and stimuli‐responsiveness) have emerged, these materials have expanded their applications in energy harvesting/storage,^[^ ^2^ ^]^ dynamic covalent polymers,^[^ ^3^ ^]^ porous materials,^[^ ^4^ ^]^ biomedical/tissue engineering,^[^ ^5^ ^]^ etc.

Among different synthesis methods (e.g., step‐growth polymerization, vulcanization, and chain‐growth polymerization), chain‐growth polymerization is one of the most commonly used mechanisms for the fabrication of polymer networks. In the chain‐growth polymerization, new monomer units (typically olefinic monomers) are added to the propagating polymer chains in a chain reaction. Addition of multivinyl monomers (crosslinkers) results in the covalent bonding of two or more propagating polymer chains, which ultimately leads to the formation of crosslinked polymer networks. Radical polymerization and reversible deactivation radical polymerization (RDRP), as subclasses of chain‐growth polymerization, are widely used in polymer network synthesis due to their broad monomer scope and functional group tolerance, which are discussed further in this review. It is worth mentioning that there are other chemistries that have been used in polymer network synthesis such as thiol‐ene,^[^ ^36^ ^]^ Michael‐addition reactions^[^ ^37^, ^38^ ^]^ and azide−alkyne cycloaddition^[^ ^39^ ^]^ which have not been covered in this review.

## 3 What Are the Benefits of Using RDRP Techniques in the Polymer Network Synthesis?

The conventional radical polymerization, which is by far the most frequently used method for synthesizing polymer networks, offers limited control over chain growth and thus final architecture of the polymer networks (Figure 1a). Structural heterogeneity in conventionally formed polymer networks is due to: i) the slow rate of activation relative to propagation and unavoidable termination, which results in little control over molecular weight and dispersity of the polymer chains and consequently leads to the formation of nanogels and clusters in the pregelation phase, and ii) the rapid chain growth combined with slow segmental relaxation in the post‐gelation period.^[^ ^6^, ^38^, ^40^, ^41^ ^]^ As well as this “microscale” heterogeneity, the higher pregelation viscosity and earlier gel point found in conventional radical polymerizations will lock in “macroscale” inhomogeneities, resulting from spatial or temporal variations in the initiation rate.

**Figure 2:**
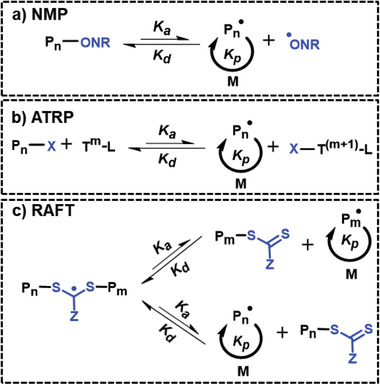
Simplified mechanisms of activation–deactivation equilibria in RDRP. a) Nitroxide‐mediated polymerization, (NMP). b) Atom transfer radical polymerization (ATRP). and c) Reversible addition–fragmentation chain transfer (RAFT) polymerization. P: polymer chain, P*_n_*•, propagating radical species, X: halides (e.g., Br, Cl), T: transition metal, L: complexing ligand and Z: reactivity‐modifying group, M: monomer.

Tuning the architecture of polymeric networks is not feasible in the postgelation phase once solids are formed and flow is limited. This means reducing the kinetic chain length and subsequently, prolonging the time to gelation allows the polymer strands to form more homogeneous architecture. This can be achieved by using different techniques such as thiol‐ene chemistry^[^ ^42^, ^43^ ^]^ or use of addition fragmentation chain transfer^[^ ^44^, ^45^ ^]^ (these systems are not covered in this review). The use of RDRP processes enables reversible equilibrium that typically lowers the concentration of propagating radicals and thereby reduces the number of termination events during the pregelation stage. Moreover, the high activation rate relative to the propagation rates of RDRP processes provides a relatively homogeneous distribution of dormant branched polymers in the early stages of network formation—these network fragments can diffuse before they are reactivated, preventing the formation of densely‐crosslinked network clusters and producing a more homogeneous network (Figure 1b). The ability to reversibly deactivate the growing polymer chains can be obtained by: i) dissociation‐combination mechanism using alkoxyamine bonds in nitroxide‐mediated polymerization, (NMP)^[^ ^8^ ^]^ (**Figure** **2**a), ii) atom transfer using alkyl‐halogen or alkyl‐pseudohalogen bond in atom transfer radical polymerization (ATRP)^[^ ^9^ ^]^ (Figure 2b), and iii) degenerative chain transfer using the alkyl‐sulfur bond of a dithioester, trithiocarbonate (TTC), dithiocarbamate, or xanthate chain transfer agent in reversible addition–fragmentation chain transfer (RAFT) polymerization^[^ ^10^ ^]^ (Figure 2c). There are numerous instances of polymer networks produced using RDRP techniques, namely NMP,^[^ ^46^, ^47^, ^48^ ^]^ ATRP,^[^ ^49^, ^50^, ^51^, ^52^ ^]^ and RAFT polymerization.^[^ ^41^, ^53^, ^54^, ^55^ ^]^ In most of these systems, RDRP agents used to homogenize the growth of chains, and hence slow the gelation. In copolymerization, where crosslinks are provided by difunctional monomers. Networks prepared by RDRP, whether by AMRP,^29‐31^ ATRP,^33‐34^ or RAFT,^36,38^ show behavior much more consistent with classical gelation theory^[^ ^56^ ^]^ than the more heterogeneous gels prepared by conventional radical polymerization. More rarely, RDRP‐derived end‐groups have been used as sources of crosslinks. For instance, Ida et al. used RAFT polymerization of *N*‐isopropylacrylamide to generate well‐defined spacers which terminal TTC end groups. These end‐groups were then converted to activated esters with *N*‐hydroxy succinimide, which were reacted with multifunctional amines to create a well‐defined polymer network.^[^ ^54^ ^]^ It should be emphasized that although RDRP techniques are used for generating polymer chains of controlled structure, molecular weight and architecture, using these techniques in polymer network synthesis neither allow direct control over the distance between crosslinking points, nor direct control of pore sizes.^[^ ^53^ ^]^ In addition to the benefits of using RDRP for the synthesis of more homogenous covalently crosslinked networks, one of the greatest opportunities provided by these techniques is the production of networks containing preserved initiating sites that can be used for posttransformation purposes. This allows modification of the shape, structure, functionality, physiochemical and mechanical properties of an already formed network in a postsynthetic stage (refer to Section 5).

## 4 Photoactivation Mechanisms in PhotoRDRP. What Does Light Offer?

**Figure 3:**
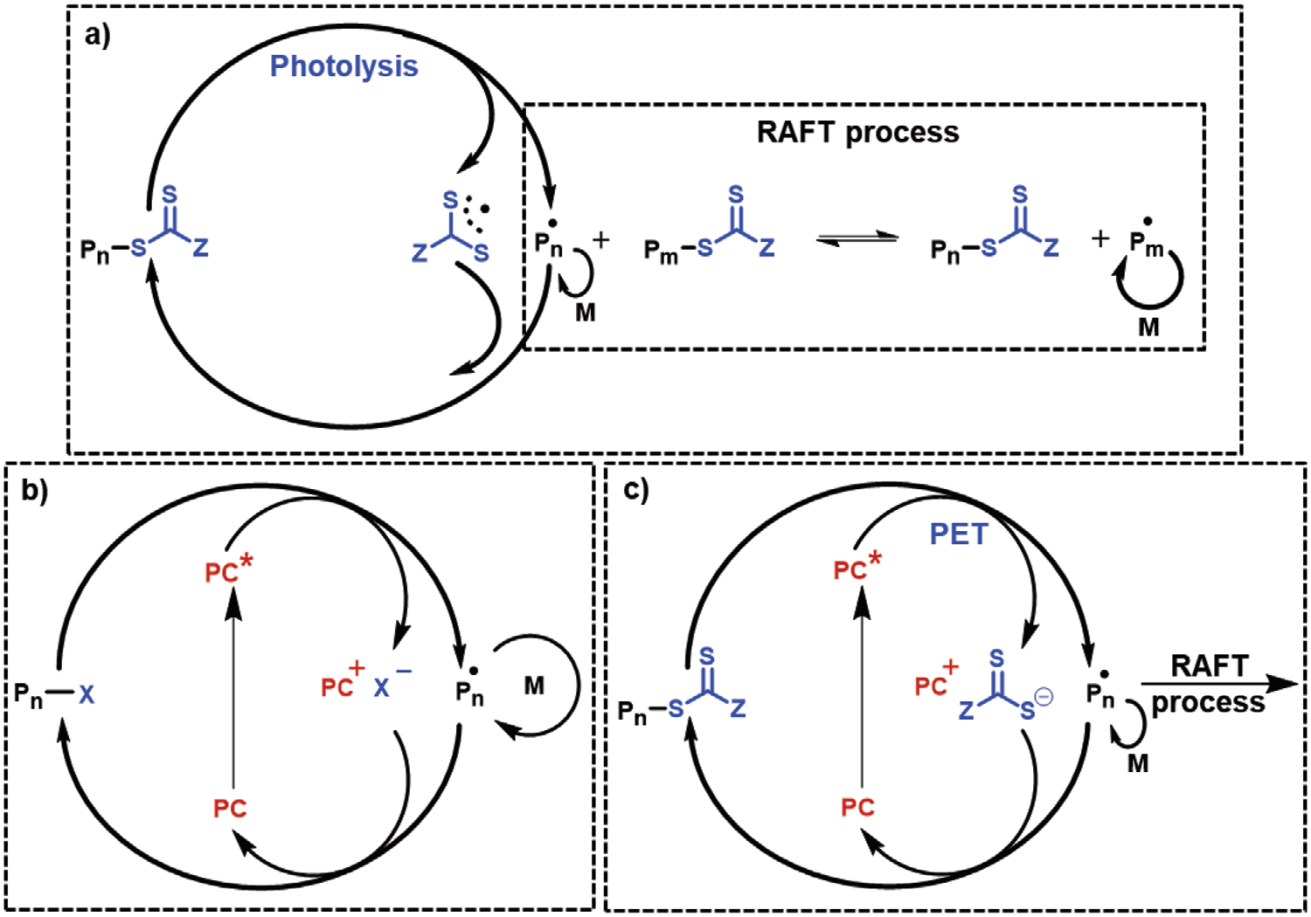
Representative example mechanisms for photoRDRP. a) Photoiniferter polymerization. b) Photoactivated atom‐transfer radical polymerization (photoATRP). c) Photoinduced electron/energy‐transfer reversible addition–fragmentation chain‐transfer (PET‐RAFT) polymerization. PC: photocatalyst; P*_n_*•, propagating radical species; M, monomer.

Early RDRP systems were typically reliant on thermal initiators to generate radicals. However, as these systems evolved throughout the years, a variety of stimuli, such as electrochemical, ultrasound and light have been exploited to reduce the dependence on elevated temperature. Light has been the preferred stimulus for the synthesis of advanced materials as it offers higher degrees of spatial and temporal controls over the polymerization.^[^ ^11^, ^12^, ^13^ ^]^ It is worth noting that type I and type II photoinitiators have been extensively exploited as radical sources to initiate radical polymerization and 3D printing systems.^[^ ^25^, ^57^ ^]^ Type I photoinitiators are single molecules capable of generating radicals directly upon bond cleavage under light exposure. In contrast, type II systems are dependent on the interaction of two components, a sensitizer and a coinitiator, for the formation of radicals.^[^ ^25^ ^]^ Although there are some examples of using photoinitiators in RDRP systems,^[^ ^14^ ^]^ the conventional photoinitiators show negligible or poor control over the reversible activation−deactivation processes. In the recently developed photoRDRP systems that have been developed more recently, not only is the activation step photocontrolled, but all of the subsequent steps (including the reversible deactivation step) can be regulated by external light source via interacting with the RDRP agents.^[^ ^58^ ^]^ PhotoRDRP can be conducted through unimolecular photoexcitation and cleavage processes. For instance, Blinco and co‐workers reported a photoNMP system using azaphenalene‐based alkoxyamine which could undergo C—O bond cleavage under UV (ultraviolet) light exposure to generate active propagating radicals and mediate polymerization.^[^ ^59^ ^]^ In a similar manner, thiocarbonylthio compounds (RAFT agents) can undergo C—S bond cleavage—via a thiocarbonyl *π* → π* transition upon UV light exposure^[^ ^60^ ^]^ or via n → π* transitions upon visible light exposure^[^ ^61^, ^62^ ^]^—and serve as photoinduced initiator‐transfer agent terminators (photoiniferters) in a radical polymerization (**Figure** **3**a).^[^ ^14^, ^21^, ^61^, ^63^, ^64^, ^65^ ^]^

PhotoRDRP systems can be also performed via photoredox catalysis.^[^ ^14^, ^17^, ^66^, ^67^, ^68^, ^69^, ^70^ ^]^ Photoredox catalysts are most often transition metal‐based catalysts, though they may be organic compounds with strong chromophores. These light‐driven catalysts have exceptional photochemical properties with strong visible light absorption, relatively long‐lived excited states, and suitable redox potentials.^[^ ^11^, ^71^ ^]^ Through either an oxidation or a reduction cycle,^[^ ^72^, ^73^, ^74^ ^]^ photoredox catalysts can generate radical species and initiate RDRP systems,^[^ ^14^, ^15^, ^16^, ^17^, ^18^, ^75^ ^]^ such as photoATRP^[^ ^15^, ^76^, ^77^, ^78^ ^]^ and photoRAFT^[^ ^12^, ^13^, ^66^, ^79^, ^80^, ^81^, ^82^, ^83^ ^]^ polymerization. As an early example of photoATRP, Fors and Hawker reported the use of a photoredox catalyst for controlling an ATRP‐type polymerization under visible light irradiation.^[^ ^11^ ^]^ In this system, an excited photocatalyst (Ir^III^*) species (which was generated upon exposure to a fluorescent lamp) could reduce an alkyl halide initiator to afford an alkyl radical (capable of initiating polymerization) and a highly oxidizing Ir^IV^ complex. This oxidizing complex could easily react with the propagating radical and thereby close the catalytic cycle, affording neutral Ir^III^ species in the ground state and a dormant halide‐capped chains.^[^ ^11^ ^]^ This cyclic process upon visible light irradiation enabled an externally regulated “on and off” system which was not achievable in conventional ATRP processes (Figure 3b).

Redox‐active catalytic species can also initiate and control the activation–deactivation equilibrium of RAFT polymerization by interacting with the thiocarbonylthio RAFT agents. This process, also referred to as photoinduced electron or energy transfer RAFT (PET‐RAFT), is based on electron or energy transfer from an excited catalyst to a RAFT agent, resulting in the cleavage of the weak C—S bonds and generation of active radical species and a stabilized RAFT agent fragment.^[^ ^66^, ^79^, ^84^, ^85^ ^]^ While recombination of these species would terminate the cyclic process, this cycle can be repeated as long as the light source is on (Figure 3c). Similar to the photoATRP systems, PET‐RAFT polymerization can be also regulated by controlling the spatial and temporal distribution of light. PET‐RAFT systems showed compatibility with a wide range of RAFT agents, monomers, and solvents, while use of a variety of catalysts (depending on their absorbance and catalytic activity) enabled PET‐RAFT polymerization under a wide range of visible or NIR light irradiation.^[^ ^66^, ^82^ ^]^ Undeniably, one of the utmost opportunities provided by PET‐RAFT is the oxygen tolerance of the polymerization systems, unlike conventional RAFT systems which tend to be inhibited by oxygen.^[^ ^86^, ^87^, ^88^, ^89^, ^90^ ^]^ Among different strategies that allow oxygen tolerant RDRP systems, such as polymerizing through oxygen,^[^ ^89^, ^91^, ^92^ ^]^ enzyme mediated deoxygenation^[^ ^93^, ^94^ ^]^ and the consumption of molecular oxygen via a PET process;^[^ ^81^, ^95^, ^96^, ^97^ ^]^ the latter approach is widely used in RAFT systems. PET process initiated from photoredox catalysts, such as Ir(ppy)_3_,^[^ ^66^ ^]^ Ru(bpy)_3_Cl_2_,^[^ ^16^ ^]^ zinc tetraphenylporphine (ZnTPP),^[^ ^13^, ^67^, ^98^ ^]^ and pheophorbide a^[^ ^81^, ^82^ ^]^ can convert triplet oxygen into a reactive oxygen species which can be trapped by a suitable quenching species and therefore enable oxygen tolerant systems. Organic dyes such as eosin Y (EY) have been also used as a PET catalyst in both solution^[^ ^12^ ^]^ and bulk^[^ ^99^ ^]^ RDRP systems for preparing linear polymers in presence of oxygen.^[^ ^12^, ^79^, ^99^, ^100^, ^101^ ^]^

**Figure 4:**
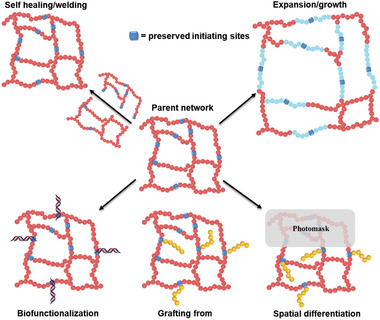
Typical examples of postsynthetic modifications of parent crosslinked networks containing initiating sites to fabricate chemically and mechanically differentiated networks. Note: These schemes are simplified, and the initiating sites can be located at the chain end, within the main chain/strand and at the branching points depending on the technique used.

The photoinducibility feature of RDRP systems has provided new possibilities in the field of advanced material. In the last decade, several papers have reported the exploitation of photoRDRP in a variety of contexts such as, nanotechnology, surface modification, flow chemistry, precision polymerization, etc.^[^ ^14^, ^15^, ^16^, ^17^, ^18^ ^]^ PhotoRDRP systems have also been exploited in both synthesis and postsynthesis modifications of polymer networks (refer to Section 5). Taking advantages of the opportunities that (photo)RDRP‐based networks can offer, numerous potential modifications of crosslinked networks have been realized; for instance self‐healing/welding,^[^ ^21^, ^102^ ^]^ grafting polymer side chains,^[^ ^103^ ^]^ expansion of network structure,^[^ ^21^, ^104^ ^]^ biofunctionalization,^[^ ^105^ ^]^ and spatial differentiation^[^ ^106^ ^]^ (**Figure** **4**). Moreover, these unconventional possibilities gave birth to the development of novel concepts of STEM gels,^[^ ^19^ ^]^ LAM,^[^ ^20^ ^]^ and PET‐PNs.^[^ ^21^ ^]^ In the next section, the evolution of transformable crosslinked networks based on (photo)RDRP systems will be discussed in detail.

## 5 Applications of (Photo)RDRP in Transformable Polymer Networks

### 5.1 Structurally Tailored and Engineered Macromolecular (STEM) Gels

**Figure 5:**
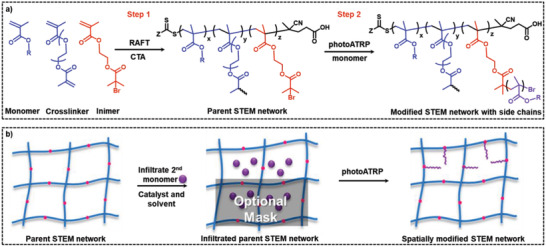
a) Synthesis of a parent STEM network by thermally initiated RAFT polymerization using a methacrylate monomer, crosslinker, and inimers (step 1); postsynthetic modification of parent STEM network after infiltrating monomer and catalyst followed by photoATRP from inimers to graft polymer side chains (step 2). b) Schematic presentation of monomer and catalyst infiltration of parent STEM network followed by spatially grafting polymer side chains from inimers using photoATRP to form spatially differentiated STEM network. Reproduced with permission.^[^ ^106^ ^]^ Copyright 2018, American Chemical Society.

The concept of STEM gels was introduced by Matyjaszewski and co‐workers, which was targeted to the fabrication of functional networks capable of undergoing postsynthetic transformation.^[^ ^19^ ^]^ The inspiration behind the development of this concept was biological stem cells which have the ability to evolve to specialized cells under specific condition or environment. In an early example, 3D ordered macroporous hydrogels with hydroxyl functional groups were synthesized via ATRP, which were subsequently served as parent STEM platforms for the formation of different multifunctional materials from an identical network. In particular, ATRP initiators were conjugated onto the surface of the hydrogels (via reaction between hydroxyl groups and *α*‐bromoisobutyryl bromide), enabling surface‐initiated ATRP of poly(*N*‐isopropylacrylamide) to form temperature‐responsive materials.^[^ ^105^ ^]^ In another study by the same group, a dual RDRP system was utilized to prepare STEM gels.^[^ ^106^ ^]^ RAFT polymerization was used to prepare parent STEM networks containing latent ATRP initiator sites (inimers—**Figure** **5**a). The use of RAFT enabled the preparation of more ordered networks compared to the conventionally formed systems. After the infiltration of parent gels with a solution containing second monomer, catalyst, and solvents, side polymer chains with predetermined lengths were grown from the inimers upon UV light exposure. Use of photoATRP enabled spatiotemporal control over the postsynthetic modification process to prepare spatially differentiated daughter materials (Figure 5b). For example, hydrophobic networks prepared with *n*‐butyl methacrylate monomer were modified to amphiphilic ones upon grafting from hydrophilic chains using 2‐(dimethylamino)ethyl methacrylate and hydroxyethyl methacrylate monomers.^[^ ^106^ ^]^

**Figure 6:**
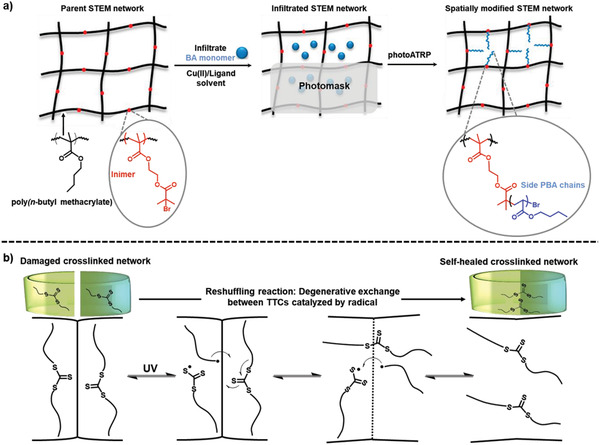
a) Procedure for postsynthetic modification of a parent STEM network by infiltrating BA monomer and catalyst and subsequent photoATRP to graft side chains from the inimers. Using spatial control, single‐piece STEM with hard or soft domains can be synthesized. Reproduced with permission.^[^ ^103^ ^]^ Copyright 2018, American Chemical Society. b) An illustration of how the TTCs undergo degenerative exchange reaction and enable self‐healing of damaged materials under UV light irradiation.

**Figure 7:**
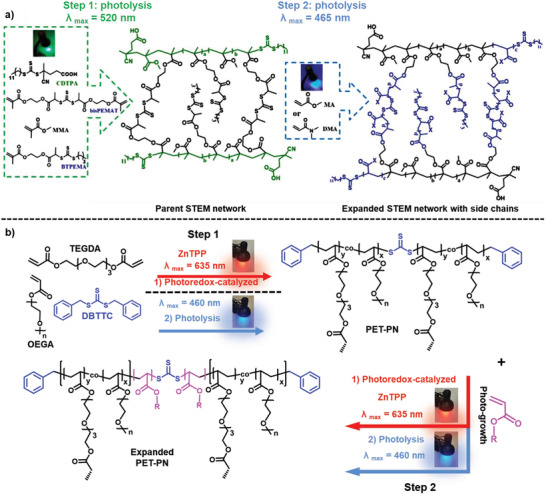
Synthesis and postsynthetic modification of crosslinked networks using photoRAFT: a) dangling and expandable parent STEM network crosslinked using TTC dimethacrylate crosslinker (bis[(2‐propionate)ethyl methacrylate] trithiocarbonate (bisPEMAT) in the presence of RAFT inimer (2‐(2‐(*n*‐butyltrithiocarbonate)‐propionate)ethyl methacrylate (BTPEMA) and RAFT agent (4‐cyano‐4‐[(dodecylsulfanylthiocarbonyl) sulfanyl] pentanoic acid (CDTPA) before modification to yield expanded STEM network with dangling side chains. Reproduced with permission.^[^ ^104^ ^]^ Copyright 2019, Royal Society of Chemistry. b) Reaction scheme of the light‐induced RAFT polymerization for the formation of a PET‐PN by either (1) photoredox catalysis (approach one) or (2) direct photolysis (approach two) mechanisms; and the subsequent photogrowth schematic through a light‐induced polymerization by either (1) photoredox catalysis (approach one) or (2) direct photolysis (approach two) mechanisms. Reproduced with permission.^[^ ^21^ ^]^ Copyright 2019, American Chemical Society.

Mechanically distinct materials can be also produced using STEM gels.^[^ ^103^ ^]^ For example, a hard STEM gel was synthesized via a RAFT copolymerization of (meth)acrylate monomers (e.g., *n*‐butyl methacrylate), di(meth)acrylate crosslinkers (e.g., poly(ethylene glycol) dimethacrylate), and (meth)acrylate ATRP initiators (e.g., 2‐hydroxyethyl methacrylate‐iBBr). This STEM gel was then modified to a soft elastomer by grafting poly(*n*‐butyl acrylate) (PBA) side chains from the latent ATRP initiator sites (**Figure** **6**a). Using this approach, a single‐piece material with distinct mechanical properties suitable for soft robotics was obtained.^[^ ^103^ ^]^ In order to transform a STEM gel into a soft, elastomeric and “non‐tacky” network, a semifluorinated poly(octafluoropentyl acrylate) was grafted from the inimers via a photoATRP process.^[^ ^107^ ^]^ In another study, a photoRAFT process was used to synthesize STEM networks containing TTC units which allowed several postsynthetic modification pathways: i) grafting from the TTC units present in the STEM networks, resulting in polymerization of dangling side chains, ii) monomer addition within the structure of the networks initiated from the TTC units of a RAFT crosslinker, resulting in the expansion/growth of the networks, and iii) a combination of both approaches resulting in both growing polymer side chains and expansion of networks (**Figure** **7**a). The networks that were initially synthesized using a RAFT crosslinker could facilitate changes in the architecture of the parent networks, while networks fabricated using conventional crosslinkers enabled the polymerization of dangling side chains (via a grafting from polymerization).^[^ ^104^ ^]^ Incorporation of TTCs or allyl sulfides within the strands of crosslinked networks to endow transformability via reversible radical reactions is elaborated in more detail in the next section.

### 5.2 Photoexpandable/Transformable‐Polymer Networks (PET‐PNs) and Living Additive Manufacturing (LAM)

One possible approach to endow transformability in the crosslinked networks is by incorporation of light‐responsive molecules within their structures. In particular, species capable of addition–fragmentation chain transfer (AFT) reactions, such as TTCs and allyl sulfides can be integrated into multifunctional monomers for direct incorporation throughout crosslinked polymer networks.^[^ ^108^, ^109^ ^]^ The addition of an AFT‐capable species within a cross‐linkable monomer enables forming a network with reversible covalent bonds. This enables unconventional properties of crosslinked networks such as plasticity,^[^ ^108^ ^]^ stress relaxation,^[^ ^108^ ^]^ actuation,^[^ ^110^ ^]^ (self)healing upon damage,^[^ ^28^ ^]^ and reprocessability/recyclability.^[^ ^20^, ^25^ ^]^ For example, Bowman and co‐workers introduced an allyl sulfide within the strands of a crosslinked network to enable plasticity and equilibrium shape changes. In their approach, residual photoinitiators (e.g., Irgacure 784 and Irgacure 819) present within the networks were exposed to light to generate radicals. These radicals were diffused through the network via the AFT reactions of the allyl sulfide functionalities. Consequently, polymer backbones within a covalently crosslinked network were repeatedly cleaved and reformed. These reversible cleavage and reformation reactions facilitated chain/strand rearrangement, resulting in a less stressed conformation.^[^ ^108^ ^]^ Since the reversible radical reaction does not consume functional groups, the sequential cleavage and reformation processes can be repeated as long as radicals are generated. In their study, a ring opening 2‐methyl‐7‐methylene‐1,5‐dithiacyclooctane monomer was employed to introduce the reversibly cleavable allyl sulfide functionalities throughout the polymer backbone.^[^ ^108^ ^]^ In addition to these types of monomers, Bowman and co‐workers proposed the use of crosslinking monomers such as di(meth)acrylates that contain linear addition–fragmentation functionalities.^[^ ^108^, ^109^ ^]^ For example, a TTC dimethacrylate monomer, capable of undergoing a RAFT polymerization, was used in a dental resin formulation to create a composite with a reduced stress as compared to the conventional dimethacrylate‐based dental composite. Addition of a RAFT‐capable monomer allows rearrangement of network strands during the polymerization by undergoing a bond breaking and reforming, resulting in polymerization‐induced stress reduction.^[^ ^111^ ^]^

Matyjaszewski and co‐workers also synthesized a TTC‐containing crosslinker monomer which was used to fabricate a TTC crosslinked network. A thermal radical initiator, 2,2ʹ‐azobis(isobutyronitrile) (AIBN) was used to generate radicals and thereby trigger reversible bond exchange between TTC units; enabling damaged networks to undergo self‐healing.^[^ ^112^ ^]^ Rather than using a thermal stimulation, Matyjaszewski and co‐workers took advantage of the photoiniferter properties of TTC units and used UV light to trigger bond exchange reactions between TTC species within the fragments of crosslinked networks, enabling repeatable UV‐induced self‐healing process (Figure 6b).^[^ ^102^ ^]^

Direct activation/photolysis of the TTCs units under UV light exposure can initiate further polymerization as presented in Figure 3a. In this context, Johnson and co‐workers used UV light to reactivate TTC moities integrated within a crosslinked network and induce polymerization of new monomers (e.g., *N*‐isopropylacrylamide) from these units.^[^ ^113^ ^]^ This process allowed an increase in the average degree of polymerization between crosslinks, and a corresponding increased in the swelling ratios of networks. It is worth mentioning that the photogrowth process, as opposed to the solution polymerization under similar condition, did not show living behavior. This was partly attributed to the limited light penetration into the network and higher light absorbance of the TTC units closer to the surface, resulting in an uncontrolled polymerization with no evidence of living chain growth throughout the entire network.^[^ ^113^ ^]^

In the last few years, research in visible light mediated RDRP systems has gained intensive interest.^[^ ^14^, ^114^ ^]^ In addition to temporal and spatial control over polymerization process, LED (light‐emitting diodes) visible light sources are ecofriendly with low energy consumption and long lifetimes. Low intensity light sources of the visible and near infrared region also reduce the risk of photodamage to living cells and biosensitive materials, making them more suitable for bio‐related applications.^[^ ^115^, ^116^, ^117^, ^118^, ^119^, ^120^ ^]^ Taking into account the benefits of the visible light spectrum, Matyjaszewski and co‐workers incorporated a visible‐light responsive thiuram disulfide (TDS) iniferter within a covalently crosslinked polyurethane polymer network. The incorporation allowed degenerative exchange between TDS units upon blue light irradiation. The S—S bond in the TDS groups can be homolytically dissociate and generate S‐based radicals, which can react with another TDS unit and form a new TDS unit. This radical exchange process resulted in reorganization of the linking units within a crosslinked network to facilitate self‐healing of damaged polymers.^[^ ^121^ ^]^

In recent thiocarbonylthio photoiniferter systems, visible light has been employed to initiate radical polymerization via simultaneous reversible activation/deactivation and degenerative chain transfer.^[^ ^60^, ^61^, ^64^, ^121^, ^122^, ^123^, ^124^ ^]^ Matyjaszewski and co‐workers reported the synthesis of TTC‐containing networks capable of postsynthetic transformation triggered by visible light irradiation, and without the presence of external initiators/catalysts.^[^ ^104^ ^]^ Bagheri and co‐workers also reported the fabrication of photoexpandable/transformable‐polymer networks (PET‐PNs) containing TTC units. In their approach, a symmetric RAFT agent, dibenzyl trithiocarbonate (DBTTC), was employed for the formation of PET‐PNs via iniferter‐mediated radical polymerization under blue LED light (*λ* = 460 nm) irradiation. The preserved DBTTC units within the strands of networks could be reactivated to enable addition of new monomers within or on the surface of parent networks. This allowed changes in the composition of networks and an increase in the network mesh size parameters (Figure 7b).^[^ ^21^ ^]^ The monomer addition process was not uniform which was partially attributed to the use of conventional crosslinker monomers (e.g., tetra(ethylene glycol) diacrylate (TEGDA) and poly(ethylene glycol) diacrylate (PEGDA)).^[^ ^21^ ^]^ It should be mentioned that other parameters such as crosslinking density, the location/distribution of TTCs, the light exposure uniformity/penetration and the (re)activatability of the TTCs can affect the efficiency of the light‐induced monomer addition into the crosslinked networks. Instead of using conventional crosslinker monomers, TTC crosslinker monomers can be used as both a crosslinking and iniferter element to provide more uniform photogrowth process.^[^ ^104^ ^]^ For instance, Harth and Lampley synthesized a TTC crosslinker which was used to fabricate nanonetworks capable of photogrowth in a postsynthetic stage.^[^ ^125^ ^]^ Upon activation of TTC units under visible light irradiation, new monomers could be added between the crosslinks and expand the nanonetworks to reach defined sizes.^[^ ^125^ ^]^

As opposed to the photoexcitation and cleavage processes of the photoiniferters, photoredox catalysts provide a means to interact with the RDRP control agents and therefore control the reversible deactivation processes.^[^ ^13^, ^16^, ^81^, ^82^ ^]^ In the context of polymer networks, photoredox catalyzed polymerization was also used for both fabrication and post‐transformation of networks. For example, Johnson and co‐workers used a 10‐phenylphenothiazine (PTH) photocatalyst to interact with the TTC units embedded within a crosslinked network and enable new monomers addition into a parent network.^[^ ^20^ ^]^ This concept was introduced as living additive manufacturing (LAM), in which an initially fabricated network could be transformed to provide a wide range of chemically and mechanically differentiated daughter networks.^[^ ^20^, ^126^ ^]^ Lampley and Harth also employed PTH photoredox catalyzed polymerization to initiate monomer addition from the TTC units embedded within crosslinked nanonetwork, enabling precise alteration in size and architecture of networks.^[^ ^125^ ^]^ Bagheri et al. also took advantage of photoredox catalyzed polymerization for both fabrication and subsequent modification of polymer networks.^[^ ^21^ ^]^ In their approach, a ZnTPP catalyst was excited under red LED light (*λ*_max_ = 635 nm, 0.7 mW cm^‐2^) to interact with DBTTC units and mediate PET‐RAFT polymerization of a diacrylate monomer to form crosslinked networks. After infiltration of new monomers (i.e., oligo(ethylene glycol) methyl ether acrylate) and a ZnTPP catalyst within the primary networks, postsynthetic monomer addition into the networks were realized in a second PET‐RAFT process.^[^ ^21^ ^]^ In the polymer networks which are formed using R—SC(=S)Z RAFT agents, the preserved functionalities can be used for surface‐initiated polymerization. Where R—SC(=S)—S—R RAFT agents or Z‐connected multi‐RAFT agents are used, the RAFT functionalities will be present in the core and connect the arms of the RAFT‐derived polymer chains which makes the postsynthetic transformation more complex.^[^ ^53^ ^]^ All the above‐mentioned studies demonstrated the use of photoRDRP in the fabrication of transformable polymer networks. The implementations of photoRDRP in practical 3D printing processes have been recently realized.^[^ ^22^, ^23^, ^24^ ^]^

## 6 Printing Using Photopolymerization

Printing of 3D materials capable of postprinting transformation can open up new possibilities for advanced materials. It can be envisaged that if a printed material requires modification in its structure, and physiochemical and mechanical properties, postprinting modification could be a more viable approach rather than fabrication of a new object. Moreover, 3D printing of materials with reprocessability can be of a great importance for highly valuable and sensitive materials used in tissue engineering and drug delivery. There are several examples of 3D printed shape memory polymers or in a broader context “4D printing” (with the fourth dimension being time) which have showed controllable shape and/or property transformations of printed materials in response to an external stimulus such as water, temperature, touch, shear, pH, and light.^[^ ^28^, ^29^, ^30^, ^31^, ^32^, ^127^ ^]^ Such changes can be achieved by incorporation of stimuli‐responsive materials into appropriate choice of printing materials.^[^ ^27^, ^128^, ^129^ ^]^ 3D printed materials containing reactivatable RDRP agents can be also categorized as a new generation of 4D printing with additional abilities (e.g., the ability of post‐printing polymerization initiated from the residual RDRP functionalities).

Before discussing the application of RDRP systems to 3D printing, we provide a brief overview of the common 3D printing techniques via photopolymerization such as stereolithography (SLA),^[^ ^130^ ^]^ digital light processing (DLP),^[^ ^131^, ^132^ ^]^ and continuous liquid interface production (CLIP).^[^ ^133^ ^]^ In general, 3D printing technology offers a programmable pathway for the fabrication of customized and on‐demand 3D networks with complex shapes and functionalities, and without wasting excess materials. These techniques rely on computer‐assisted design 3D models, which are translated into physical 3D networks in a layer by layer fashion.^[^ ^134^, ^135^, ^136^, ^137^ ^]^ The photopolymerization‐based 3D printing is based on using monomers/oligomers in a liquid state that can be cured to form crosslinked polymer networks upon exposure to light.^[^ ^57^, ^138^ ^]^ SLA is a method in which lasers (typically emitting in the UV range) are utilized to initiate photopolymerization and crosslinking of liquid resin to print solid layers one on top of the other.^[^ ^139^ ^]^ Materials produced using SLA have been used in a variety of contexts such as tissue engineering.^[^ ^140^ ^]^ responsive hybrid materials,^[^ ^141^ ^]^ and drug delivery systems.^[^ ^142^ ^]^ In contrast to the SLA systems which operate via point‐by‐point laser exposure from top, DLP systems use LED (with a wide range of wavelengths from deep UV to visible) to induce the crosslinking of photocurable resins by illuminating each layer all‐at‐once and from the bottom of the resin vat.^[^ ^131^, ^143^, ^144^, ^145^ ^]^ This allows reduced printing times while maintaining high manufacturing accuracy.^[^ ^131^ ^]^ DLP technique has been widely used for manufacturing complex objects such as highly stretchable photopolymers,^[^ ^146^ ^]^ reprocessable thermosets,^[^ ^147^ ^]^ etc.^[^ ^137^, ^148^, ^149^, ^150^ ^]^ We urge the readers to refer to recent reviews for more details.^[^ ^25^, ^145^ ^]^

**Figure 8:**
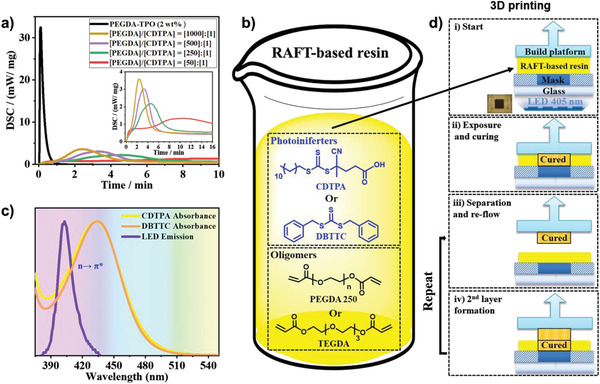
a) Photodifferential scanning calorimetry (photo‐DSC) plots of different RAFT‐based formulations containing CDTPA iniferter. b) RAFT‐based visible light‐curable formulations containing different TTCs: CDTPA and DBTTC; and crosslinkers: PEGDA 250 and TEGDA. c) UV–vis absorption spectra of TTC units and the emission spectrum of LED light source of 3D printer, showing emission‐absorption overlap (both spectra are normalized); and d) sequential steps of 3D printing using a modified bottom‐up DLP printer equipped with LED lights (*λ*_max_ = 405 nm, 1.8 mW cm^–2^) at room temperature and under nitrogen atmosphere. Reproduced with permission.^[^ ^22^ ^]^ Copyright 2020, Royal Society of Chemistry.

**Figure 9:**
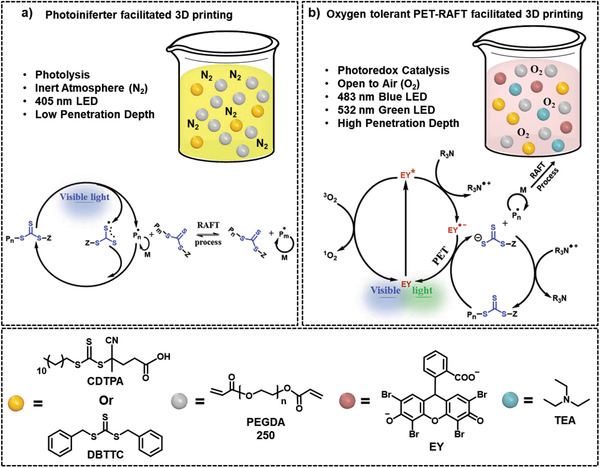
Comparison of methods used to realize RAFT‐based 3D printing. a) Using photolysis in the absence of air. Proposed mechanism of a visible light induced RAFT iniferter approach as previously reported by Qiao's group^[^ ^62^ ^]^ and Boyer's group.^[^ ^61^ ^]^ b) Using photoredox catalysis conducted fully open to air. Proposed photopolymerization mechanism under visible light irradiation where the excited‐state EY is reduced by a tertiary amine, leading to a reductive PET‐RAFT process. This mechanism has been previously reported in both solution^[^ ^12^ ^]^ and bulk^[^ ^99^ ^]^ polymerization for preparing linear polymers.^[^ ^100^ ^]^

Radical polymerization has generally been one of the mechanisms used in vat photopolymerization. As extensively described in Section 3, the strands of crosslinked networks produced using conventional radical polymerization cannot be reactivated for posttransformations. The application of photoRDRP to 3D printing can render postprinting polymerization a reality by introducing dormant functionalities into the printed object. This area of research was first reported by Bagheri and Jin in collaboration with Boyer.^[^ ^22^, ^23^, ^24^ ^]^ Inspired by the development of photoRDRP‐based networks, Bagheri et al. collaborated with Boyer's group on the first examples of 3D printing process facilitated by a photoiniferter (RAFT) system.^[^ ^22^ ^]^ In their approach, a photocurable formulation containing a TTC iniferter (such as CDTPA or DBTTC) and a crosslinker monomer (such as PEGDA or TEGDA) was first prepared in the complete absence of exogenous initiators/catalysts and solvent. The photoreactivity of the TTC‐containing formulation was compared to that of formulation containing traditional photoinitiator, such as diphenyl (2,4,6‐trimethylbenzoyl) phosphine oxide (TPO)). The gelation point was delayed from few seconds to few minutes in presence of photoiniferters (**Figure** **8**a,b).^[^ ^22^ ^]^ This inhibition in the photopolymerization rate was partly attributed to the slow activation rate of iniferters (mechanism presented in **Figure** **9**a) as well as possible trace of oxygen in the system.^[^ ^10^ ^]^

**Figure 10:**
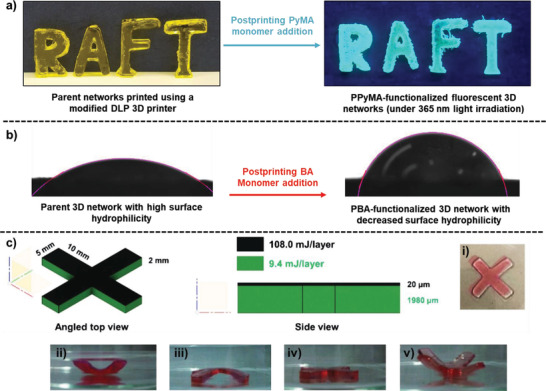
a) Optical image of an initially printed “RAFT” word and its subsequent modified network after PyMA insertion. Reaction scheme of the postprinting PyMA insertion. Polymerization of PyMA or in another word, PyMA monomer insertion into the printed objects were performed at 70 °C for 24 h. Reproduced with permission.^[^ ^22^ ^]^ Copyright 2020, Royal Society of Chemistry. b) Water contact angles of an initially printed object and its subsequent modified network after BA insertion. Reaction scheme of the postprinting BA insertion. Polymerization of BA (BA insertion into the printed objects) was performed under blue LED light, without presence of external initiators or catalysts. Reproduced with permission.^[^ ^23^ ^]^ Copyright 2020, American Chemical Society. c) Swelling and desolvation induced actuation of a material 3D printed with spatially resolved light doses. Designed geometrical properties of cross; i) top view of swollen cross geometry (layer exposed to higher light dose on bottom of object); ii) cross with layer exposed to higher light dose on the bottom, after 5 mins in water; iii) flipped swollen cross before dehydration (layer exposed to higher light dose on top); iv) cross after 80 s of dehydration; v) cross after 7 mins of dehydration. Reproduced with permission.^[^ ^24^ ^]^ Copyright 2019, John Wiley and Sons.

The layer by layer printability of the iniferter containing formulations was demonstrated using a modified DLP 3D printer equipped with 405 nm LED lights (Figure 8d).^[^ ^22^ ^]^ It is known that carbon‐centered radicals generated in photoiniferter polymerization can react with molecular oxygen to form peroxy radical and hydroperoxides which are not reactive to reinitiating polymerization.^[^ ^66^, ^86^, ^87^, ^88^, ^89^, ^90^ ^]^ To avoid retardation, 3D printing process was conducted inside a glovebox under inert atmosphere.^[^ ^22^ ^]^ Although the presence of iniferters significantly slowed down the 3D printing speed, the use of iniferter‐based formulation was a compromise to enable printing of 3D materials containing dormant iniferter units. In a postprinting stage, a 3D printed object (“RAFT” word) was first subjected to a medium containing a fluorescent 1‐pyrenemethyl methacrylate (PyMA) monomer^[^ ^151^, ^152^, ^153^, ^154^, ^155^, ^156^, ^157^ ^]^ and a thermal initiator (AIBN) in solvent. Subsequently, upon reactivation of the preserved TTC units, PyMA monomers were added within and on the surface of the “RAFT” object to afford a fluorescent pyrene‐functionalized network (**Figure** **10**a).^[^ ^22^ ^]^ Postprinting monomer addition into a highly crosslinked network formed using symmetrical RAFT agents, are complex and requires further exploration. In particular, end‐group transformation/activation of R—SC(=S)—S—R RAFT agents or “Z”‐connected bis‐RAFT agents present within crosslinked networks might lead to the cleavage of the thiocarbonylthio linkage and (partially) degrade the network structure.^[^ ^53^ ^]^ Possibly, networks formed from DBTTC units contain —C(=S) —S—CH_2_Ph ends rather than strands incorporating TTC linkages. In the networks formed from CDTPA RAFT agents, preserved chain‐ends can be reactivated to enable surface‐initiated polymerization.

The iniferter mediated 3D printing process had two major downsides: i) oxygen inhibition, which was mitigated by performing the 3D printing process under inert conditions, and ii) slow direct iniferter activation due to the low extinction coefficient of the n→ π* transition under visible light (Figure 9a). To address the limiting issues, an EY photocatalyst and a sacrificial triethylamine reducing agent were added to the initial iniferter‐based formulation, enabling an oxygen tolerant system (Figure 9b).^[^ ^23^ ^]^ The inclusion of tertiary amines into EY‐based RAFT polymerization provides more energetically favorable PET process and therefore increases the polymerization rate.^[^ ^79^, ^101^ ^]^ Upon visible light excitation, excited EY^*^ catalyst can be reduced by triethylamine (which can act as an electron donor) to produce an eosin radical anion (EY^• −^) and an amine radical cation (R_3_N^• +^).^[^ ^158^ ^]^ The reduced EY^•−^ catalyst can then transfer an electron to the RAFT agent (e.g., DBTTC or CDTPA) and return back to the ground state. Subsequently, the reduced RAFT species undergo *β*‐cleavage of the weak C—S bond to generate a RAFT stabilized anion species and a radical propagating species (P*_n_*•) capable of adding monomers and interacting with other RAFT agents to enable a RAFT process. In a EY‐based system, the active molecular oxygen can be consumed to form inactive superoxide anions by electron transfer from an eosin radical anion (EY^• −^) or anion RAFT species (Figure 9b).^[^ ^12^, ^86^, ^159^ ^]^ A modified DLP 3D printer equipped with blue (*λ*_max_ = 483 nm, 4.16 mW cm^‐2^) or green (*λ*_max_ = 532 nm, 0.48 mW cm^‐2^) LED lights was used for printing of the PET‐based formulations fully open to air.^[^ ^23^ ^]^ Use of green LED showed even higher 3D printing build speed as compared to its alike blue LED‐induced printing, which was partly attributed to a greater absorbance‐emission overlap between EY and green LED emission.^[^ ^23^ ^]^ It should be noted that Sumerlin, Boyer and co‐workers elucidated the mechanism of EY‐based PET‐RAFT linear solution polymerization, in which blue light irradiation resulted in a multiple activation mechanism and reversible‐termination steps. The use of visible light of higher wavelength (e.g., green light) enables mainly PET pathways and limits other possible activation mechanisms (e.g., photoiniferter).^[^ ^100^ ^]^

The 3D materials printed using the EY‐based formulations were also capable of undergoing postprinting modification. For example, a hydrophilic PEGDA‐based printed network was first infiltrated with a hydrophobic BA monomer and solvent. This was followed by reactivation of the preserved TTC moieties present throughout the network to add new BA monomers within or onto the surface of the parent material, producing a daughter network with increased hydrophobicity (Figure 10b).^[^ ^23^ ^]^ In parallel, Boyer and Corrigan in collaboration with Bagheri and co‐workers also exploited an oxygen tolerant PET‐RAFT system using a 2‐(butylthiocarbonothioylthio) propanoic acid RAFT agent, a water soluble erythrosin B catalyst and a triethanolamine cocatalyst to enable 3D printing of PEGDA‐based materials in aqueous solutions without prior deoxygenation and under green light irradiation (*λ*_max_ = 525 nm, 0.32 mW cm^‐2^).^[^ ^24^ ^]^ The preserved TTC units present within the printed networks were then reactivated for surface functionalization with BA monomers. Interestingly, a “cross” was printed where the first layers were exposed to a high dose of light and subsequent layers were exposed to lower dose of lights. This change of light exposure enabled a route for swelling‐ and dehydration‐induced actuation (Figure 10c).^[^ ^24^ ^]^ More recently, Boyer, Corrigan and co‐workers have investigated the effect of multi‐arm RAFT agents (1, 2, 3, 4 arms) on the mechanical properties of 3D printed objetcs. Notably, a relatively low ratio of monofunctional RAFT agent was found to concurrently enhance storage modulus and fracture toughness in comparison with . In addition, changing the concentration and functionality of RAFT agents provided control over material mechanical properties in a broad span. In another study, porphyrinic zirconium metal‐organic frameworks loaded with Zn was used as a photocatalyst to mediate a 3D printing process under green light irradiation. This system enabled stereolithography of both soft and hard materials by varying the formulations.^[^ ^160^ ^]^ The initial studies of photoRAFT‐based 3D printing have showed great promise in designing advanced transformable materials and serve as a basis for future studies.

## 7 Concluding Remarks and Future Perspectives

Polymer network is one of the most important groups of materials which finds applications in the health, energy, food, and manufacturing sectors (3D printing). Typically, photopolymerization‐based 3D printing systems rely on conventional polymerization mechanisms, such as radical polymerization or cationic polymerization, to produce such polymer networks. More recently, the introduction of photoRDRP has provided a pathway for the preparation of these gels and 3D printing of “living” materials containing dormant reactivatable species that can be used for postprinting transformations. This allows the preparation of progeny materials with chemically and/or mechanically distinguished properties stemming from one parent materials to meet the demand of advanced applications and environmental sustainability concerns. The development of RDRP over the past few decades has provided further opportunities by enabling synthesis of polymer networks with tunable architectures and site‐specific functionalities unachievable using conventional polymerization methods. In addition, photoRDRP has further contributed to the development of more advanced polymer networks with unconventional properties, (self)healing/repairability upon damage.

Although the field of RDRP‐based polymer networks is widely studied, the implementation of photoRDRP in 3D printing technology is still in the early stages of development. Low photopolymerization rate, oxygen inhibition, uncontrolled distribution, and (re)activatability of the preserved initiating sites within the structure of polymer networks may be the major limiting factors to broaden the scope of RDRP application in the 3D printing field. Therefore, conducting research in the following areas would be essential in moving this field forward: i) the development of oxygen tolerant photoRDRP system with high fast kinetics in bulk at room temperature, ii) the development of an approach to control the distribution of dormant initiating sites within the structure of highly crosslinked networks, and iii) systematic studies on the (re)activatability of the preserved initiating sites. The development of 3D printed materials with living characters can be also extended to bioprinting applications. Taken all together, the field of photoRDRP‐based polymer networks has the potential to unlock exciting new research opportunities with practical applications.

## Conflict of Interest

The authors declare no conflict of interest.
